# Editorial: Exploring the role of adaptive immunity in chronic airway respiratory diseases

**DOI:** 10.3389/falgy.2024.1446656

**Published:** 2024-06-26

**Authors:** Evangelia Fouka, Apostolos Bossios, Paschalis Steiropoulos, Konstantinos Samitas

**Affiliations:** ^1^ Pulmonary Department, Medical School, Aristotle University of Thessaloniki, “G. Papanikolaou” General Hospital, Exohi, Thessaloniki, Greece; ^2^ Division of Lung and Airway Research, Institute of Environmental Medicine, Karolinska Institutet, Stockholm, Sweden; ^3^ Karolinska Severe Asthma Center, Department of Respiratory Medicine and Allergy, Karolinska University Hospital, Stockholm, Sweden; ^4^ Department of Respiratory Medicine, Medical School, Democritus University of Thrace, Alexandroupolis, Greece; ^5^ 7th Respiratory Clinic, “Sotiria” Chest Hospital, Athens, Greece

**Keywords:** adaptive immunity, t-regulatory (Treg) cells, asthma, COPD—chronic obstructive pulmonary disease, ILC2—group-2 innate lymphoid cell

**Editorial on the Research Topic** Exploring the role of adaptive immunity in chronic airway respiratory diseases

Chronic obstructive airway diseases, mainly asthma and chronic obstructive pulmonary disease (COPD), represent a significant global health burden (1). Although these are distinct disease entities, they do share similar clinical and immunopathological characteristics. Both are characterized by persistent respiratory symptoms and airflow limitation due to airway and/or alveolar abnormalities, often caused by exposure to allergens or noxious particles and gases, pathogenic bacteria, and viruses, both of which require interventions, such as the removal of triggering factors (allergens and smoking) and treatment with inhaled bronchodilators and anti-inflammatory agents, mainly corticosteroids (2).

Adaptive immunity plays a central role in the pathology of both the diseases. In type-2 (T2) high asthma, highly specialized T-helper (Th) lymphocytes and activated B cells, in conjunction with cells of natural immunity, such as group-2 innate lymphoid cells (ILC-2), orchestrate the initiation and perpetuation of the underlying allergic and/or eosinophilic inflammation (3, 4). In T2-low asthma, the inflammatory process is mostly based on the detrimental effects of neutrophils rather than eosinophils, activated by Th-1 and 17 lymphocytes of the adaptive system along with ILC-3 cells of the innate system (5, 6). COPD pathology resembles T2-low inflammation with respect to neutrophil activation; however, the initial harmful stimuli and immunological events that lead to bronchial inflammation and emphysema are different (7).

Recent studies have attempted to elucidate how adaptive immune responses, in conjunction with the innate system, can perpetuate airway inflammation and remodeling in chronic obstructive airway diseases. A recent study by Liu et al. delves into the mechanisms connecting dietary antioxidant intake and the risk of chronic obstructive pulmonary disease (COPD), with particular emphasis on the potential role of inflammatory factors as mediators. Vitamins and other antioxidants are well-known for their genomic and non-genomic effects on both innate and adaptive immunity (8). In this context, the authors analyzed a sample of 8,257 US adults aged ≥40 years using multivariate logistic regression and mediation analysis, utilizing data from the National Health and Nutrition Examination Survey (NHANES) for the period–2007–2012. The results of this study revealed that increased carotenoid intake and a higher Composite Dietary Antioxidant Index (CDAI) were associated with a decreased risk of COPD. Moreover, the study found that inflammatory factors such as leukocytes, alkaline phosphatase, and C-reactive protein exhibited a negative correlation with CDAI levels. Based on these findings, the study suggested that higher levels of dietary antioxidants, particularly carotenoids, may aid in reducing the incidence of COPD by mediating the effects of inflammatory factors and that the CDAI could serve as a useful tool for assessing the risk of COPD.

In the study by Klein et al., the authors explored the role of regulatory T cells (Tregs) in allergic asthmatic and non-asthmatic individuals following low-dose allergen challenges using healthy individuals without allergy or asthma as controls. To assess Treg function, subjects underwent a range of tests, including expiratory flow measurements, sputum induction, and blood sampling. The key findings revealed that, in non-asthmatic allergic subjects, Tregs exhibited improved suppressive function after allergen exposure, in contrast to asthmatic subjects, where this function was not observed.

Several studies have implicated impaired Treg functionality in the pathophysiology of asthma (9, 10). Considering these results, non-asthmatic allergic individuals may develop short-term tolerance mechanisms to allergens, which are not evident in asthmatic subjects. Th-17 cells are also implicated in non-T2 responses in certain aspects of asthma and COPD. They produce IL-17, which promotes the recruitment and activation of neutrophils and contributes to airway inflammation and remodeling in both diseases. Therapies targeting the IL-17 pathway have been investigated as potential treatments for certain asthma phenotypes and are currently being considered for COPD (11). Camargo et al. examined the effects of anti-IL-17 treatment on asthma-COPD overlap (ACO) in an animal model. Mice were sensitized with ovalbumin, exposed to porcine pancreatic elastase or both, and then treated with anti-IL-17 monoclonal antibody or saline. The findings revealed that treatment reduced airway hyper-responsiveness, inflammatory cell counts in bronchoalveolar lavage fluid, and mean alveolar diameter. These results suggest that inhibiting IL-17 modulates cytokine production, extracellular matrix remodeling, and oxidative stress in ACO, providing a pathophysiological background for potential therapeutic interventions inhibiting IL-17 in both smoking asthmatics and COPD patients.

The interaction between innate and adaptive immunity is crucial in the pathogenesis of inflammatory airway diseases. In addition, Triverdi et al. used a specialized animal model to examine the impact of 17β-estradiol on the activation of ILC-2 and NF-κB in the context of asthma, particularly in women during their reproductive years. *Ε*strogen treated mice displayed less production of IL-5 and IL-13 by lung ILC-2 as compared to the controls, indicating a protective effect against T2-high inflammatory responses often observed in asthma. The findings of this study have significant implications as they provide a mechanistic background for a specific endotype of late-onset asthma involving postmenopausal women and provide insights into the protective effects of estrogen in women with asthma by suppressing ILC2, which could lead to new therapeutic targets.

The studies highlighted in this research topic collectively enhance our understanding of the intricate role that adaptive immunity plays in chronic obstructive airway diseases, along with natural immunity. Elucidating the specific immune pathways that contribute to airway disease pathogenesis and progression is the initial first step that we must be made to identify promising targets for novel therapeutic approaches that will not only alleviate symptoms but also modify the disease course.
